# Association between multimorbidity and quality of life after hip replacement surgery: analysis of routinely collected patient-reported outcomes

**DOI:** 10.1016/j.bja.2024.08.037

**Published:** 2024-11-13

**Authors:** Nicola J. Vickery, Alexander J. Fowler, John Prowle, Rupert Pearse

**Affiliations:** CCPMG, William Harvey Research Institute, Queen Mary University of London, London, UK

**Keywords:** anaesthesia, hip replacement, multimorbidity, orthopaedic surgery, patient-reported outcomes, perioperative medicine, quality of life, shared decision-making

## Abstract

**Background:**

Total hip replacement surgery is performed to improve quality of life (QoL). We explored the association between multimorbidity and change in QoL after total hip replacement.

**Methods:**

Analysis of patients included in the NHS England hip replacement Patient Reported Outcome Measures (PROMs) database with complete preoperative from 3 to 6 months postoperative EQ-5D QoL data from April 2013 to March 2018. Multimorbidity was defined as two or more chronic diseases excluding arthritis. The primary outcome measure was change in QoL using the Pareto Classification of Health Change. We compared QoL change for patients with and without multimorbidity and those with no multimorbidity using multivariable modelling. Data are presented as odds ratio (OR) with 95% confidence interval or *n* (%).

**Results:**

Of 216,191 patients, we included 178,129 (82.4%) patients with complete data. Most patients 63,327 (35.6%) were 70–79 yr of age, and 98,513 (55.3%) were women. Multimorbidity was present in 38,384 patients (21.6%). QoL improved after surgery for 149,774 (84.1%) patients, remained unchanged for 10,219 (5.7%) patients, and became worse after surgery for 7289 (4.1%) patients. QoL changes were mixed (at least one QoL domain improved and at least one deteriorated) for 10,847 (6.1%) patients. Poor QoL outcomes (unchanged/mixed/worse) were more likely for patients with multimorbidity (OR 1.53 [1.49–1.58]).

**Conclusions:**

Hip replacement surgery improves QoL. However, patients with multimorbidity are less likely to experience these benefits. Poor QoL outcomes became more frequent as the number of comorbid diseases increased. These data should inform shared decision-making conversations around joint replacement surgery.

**Editor's key points**

• Total hip replacement surgery is one of the most common surgical procedures globally, and can be associated with major mortality and morbidity.
• This analysis included patients in the NHS England hip replacement Patient Reported Outcome Measures from 2013 to 2018.
• The primary outcome measure of change in quality of life was improved after surgery for 149,774 (84.1%)patients, unchanged for 10,219 (5.7%) patients, and worse for 7289 (4.1%) patients. Poor outcomes were more likely for patients with multimorbidity.
• Although hip replacement surgery improves quality of life in the majority, a significant minority of patients with multimorbidity are less likely to experience improved quality of life.
• These data should facilitate shared decision-making conversations around joint replacement surgery in older and chronically ill patients.

Degenerative joint diseases, such as osteoarthritis, result in significant pain, disability associated with decreased range of motion, and subsequent reduction in quality of life (QoL). Major joint replacement is the treatment of choice for debilitating joint disease, and is one of the commonest performed surgeries within the UK National Health Service (NHS). Worldwide, more than one million hip replacements are performed annually,^1^ with more than 70,000 primary hip placements performed annually to relieve symptoms and improve patient's QoL.^2^^,^^3^

Joint replacement surgery is highly effective for the vast majority of patients.^1^^,^^4^ However, it is major surgery and not without resultant morbidity and mortality. Despite mostly favourable outcomes, there are high-risk patients who suffer debilitating complications, diminished QoL, or death in the postoperative period.^1^^,^^5^, ^6^, ^7^, ^8^ Identifying these high-risk patients in the context of an ageing surgical population is an important area of research.^9^ As age increases, the prevalence of chronic diseases increases.^10^ There is a growing awareness of how individual comorbidities associate with poor outcome, and how diseases combine^11^ to impact patient outcomes.

The associations between underlying chronic disease and multimorbidity (defined as the coexistence of two or more chronic conditions^12^) and subsequent QoL are unclear. We are currently unable to forecast who will have reduced disability with an improved QoL, and who will not, after hip replacement surgery. This limits shared decision-making for patients considering hip replacement surgery.

We hypothesise that patients with more chronic diseases have a reduced gain in QoL after hip replacement surgery, and that different patterns of multimorbidity are associated with different changes in QoL after surgery. We explored the risk factors that are associated with lesser gains in QoL after hip replacement surgery.

## Methods

### Study design

We used routinely collected data from the NHS England hip replacement Patient Reported Outcomes Measures (PROMs) database for this prospectively planned analysis. No research ethical approval was required for this analysis of freely available, anonymised data.^2^ We report our findings in line with Strengthening The Reporting of OBservational studies in Epidemiology (STROBE) guidance.^13^ We developed a statistical analysis plan before undertaking data analysis.

### Setting

PROMs data are collected from patients who have had specific surgeries funded by NHS England. The PROMs dataset comprises preoperative and postoperative patient perceived and patient self-reported outcomes looking at quality of care and surgical sequelae for commonly performed elective surgeries. These surgeries include all patients undergoing hip or knee replacement surgery funded by NHS England. Patients complete a questionnaire before and between 3–6 months after surgery.

### Participants

We included all patients undergoing total hip replacement who were represented in the PROMs database between April 1, 2013 and March 31, 2018. Patients are represented in PROMs if they return at least one questionnaire and have successful linkage to hospital episode statistics (HES) records.^14^

### Exposure variables

Key exposure variables collected by the PROMs questionnaires include age band (in decades from 20 yr of age), sex, 12 common chronic diseases, whether it was primary or revision surgery, preoperative PROMs including the disease-specific Oxford Hip Score (OHS), and social factors such as assistance, symptom period, performance of activities of daily living, and disability. The OHS is a validated, sensitive, and reproducible 12-item patient-reported outcome measure for use in patients undergoing hip replacement surgery. It provides a score from 0 to 48 to indicate disease severity, and is often used to guide treatment.^15^ Comorbidities were prespecified and self-reported by patients as being present or not at the time of questionnaire completion. Multimorbidity was defined as two or more self-reported chronic diseases. We excluded arthritis from the definition of multimorbidity, as this is the indication for most hip replacement surgeries.

### Primary outcome measure

The primary outcome was improvement in QoL, classified using the Pareto Classification of Health Change (PCHC). The PCHC^16^ compares preoperative and postoperative EuroQoL-five-dimensional (EQ-5D) scores and divides them into: (1) better (improvement on at least one EQ-5D dimension and no worsening in any other dimension); (2) unchanged (no change in any of the EQ-5D dimensions); (3) mixed (improved in at least one dimension and worsened in at least one other dimension); or (4) worse (worsening in at least one EQ-5D dimension and no improvement in any other dimension).^16^ The EQ-5D is a widely used, well-validated, self-reported generic measure of QoL that evaluates five health dimensions: mobility, self-care, usual activities, pain/discomfort, and anxiety/depression.^17^^,^^18^ It evaluates each health dimension on three levels as no issues (=1), slight/moderate issues (=2), and extreme/severe issues (=3).^18^ It includes a visual analogue scale (EQ-VAS), with 0 being the worst imaginable health state to 100 being the best imaginable health state, which captures a broader overall measure of health than the core five domains. We defined a change in EQ-VAS as the difference between the preoperative EQ-VAS score and the postoperative EQ-VAS score.

### Bias and missing data

The major source of bias is missing data as the outcomes are questionnaire based and reliant on the return of the questionnaires. In addition, patients who die, potentially with the highest disease burden, are not included; thus this is an analysis of those who survived. Our prespecified analysis included all patients with complete data for EQ-5D in each domain (measured as EQ-Index Profile, a summary label of five ordinal numbers for each domain) and EQ-VAS. We present the characteristics of records with missing EQ-5D data. We treated suppressed age band (∗) as its own category. Each disease is recorded as either present (1) or not reported/missing (9). We considered patients with not reported/missing values to not be suffering from the disease.

### Statistical analysis

We developed a statistical analysis plan before analysis. Datasets were downloaded electronically.^2^ Patients with missing data in EQ-VAS or EQ-Index Profile were excluded from the analysis. All independent variables were categorical in the data source and are presented as *n* (%). We calculated the frequency of pairs and triads of disease combination. The primary outcome measure was change in QoL using the PCHC by which we stratified the patient-reported data. We modelled PCHC categories as the QoL outcome using multivariable logistic regression. Independent variables included in the multivariable model were selected for biological plausibility (age bands, sex, presence of multimorbidity, OHS category, symptom period). We modelled change in EQ-VAS using a multivariable linear regression model, with *P*<0.05 considered significant. Data are presented as odds ratio (OR) with 95% confidence interval (CI) or *n* (%). Statistical analyses were conducted using R^19^ and associated packages.^20^, ^21^, ^22^, ^23^, ^24^, ^25^, ^26^, ^27^, ^28^

We repeated the core analysis excluding data on a per-domain basis as an exploratory analysis. For example, if a patient had recorded all preoperative domains apart from self-care, we excluded only that domain from analysis for that patient, with all other domains remaining eligible for analysis.

## Results

### Participants

**Fig 1:**
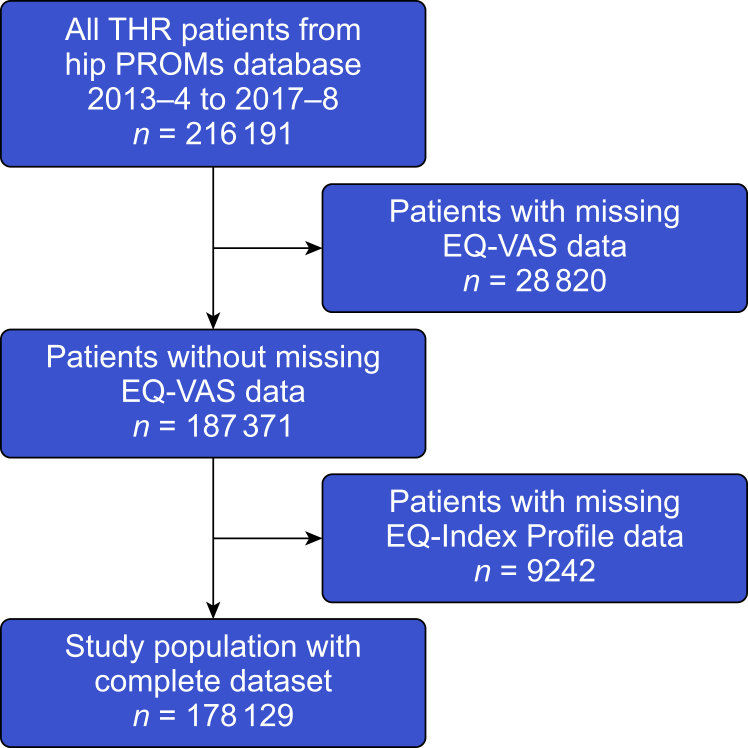
Study population flowchart. PROMs, Patient Reported Outcome Measures; THR, total hip replacement; VAS, visual analogue scale.

We identified 216,191 patients undergoing hip replacement present in the PROMs dataset during the study period. These patients represent 61.5% (216,191/351,729) of patients who underwent hip replacement surgery with an overall mortality of 0.24%, per the National Joint Registry.^29^ We excluded 38,062 (17.6%) patients who had missing data in the EQ-Index Profile or EQ-VAS; the characteristics of these patients are listed in Supplementary Table S1. There was no statistically significant difference between the complete and incomplete datasets with *P*>0.05. The final analysis set included 178,129 (82.4% of total) patients; the process of patient selection is summarised in Figure 1.

### Characteristics of the cohort

**Fig 2:**
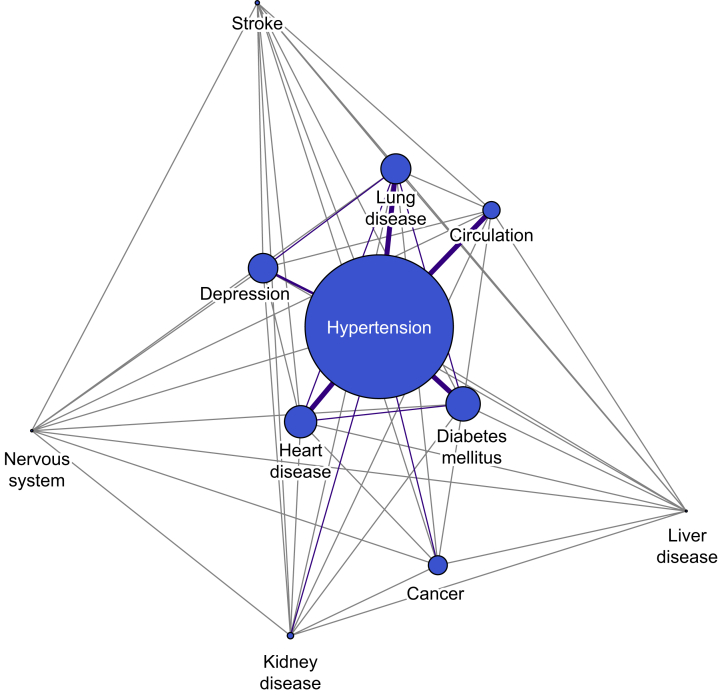
Network graph. The network plot shows interactions of all chronic diseases bar arthritis and the strength of the relationship between them. The light grey lines represent a prevalence association of less than 1%, purple lines representing a prevalence association of more than 1%, with the thicker the line showing a stronger association. The size of the comorbidity dot is the prevalence of the disease in the cohort.

Most patients were aged 70–79 yr (63,327; 35.6%), and 98,513 (59.5%) were female (Table 1). Arthritis was the most common chronic disease reported, affecting 128,464 (72.1%) patients. The four most common chronic diseases after arthritis were hypertension (68,546; 38.5%), diabetes mellitus (16,374; 9.2%), heart disease (15,773; 8.9%), and lung disease (14,642, 8.2%). Multimorbidity was present in 38,384 patients, 21.6% of the cohort. A network graph of all comorbidities (Fig. 2) shows that the most common comorbidity, hypertension, is associated with a >1% prevalence with six other comorbidities. There are also notable relationships of >1% prevalence with lung disease, heart disease, and diabetes mellitus. There were 50 pairing combinations. The most common pairs of disease were hypertension:diabetes mellitus (10,090), heart disease:hypertension (6705), and hypertension:lung disease (1644). There were 61 triad combinations, with the most common triads being heart disease:hypertension:diabetes mellitus (1759), heart disease:hypertension:lung disease (910), and hypertension:lung disease:diabetes mellitus (807) (Supplementary Tables S2 and S3).

**Table 1:** Overall cohort characteristics stratified by Pareto Classification of Health Change.

		Overall cohort		Pareto Classification of Health Change							
				Better *n* %		Unchanged *n* %		Mixed *n* %		Worse *n* %	
		178,129 (total %)		149,774	84.08	10,219	5.74	10,847	6.09	7289	4.09
Age bands (yr)											
	Not specified	12,651	7.10	10,585	83.67	680	5.38	867	6.85	519	4.10
	20–29	5	0.00	5	100.00	0	0.00	0	0.00	0	0.00
	30–39	168	0.09	124	73.81	18	10.71	17	10.12	9	5.36
	40–49	2906	1.63	2380	81.90	176	6.06	194	6.68	156	5.37
	50–59	21,103	11.85	17,655	83.66	1102	5.22	1342	6.36	1004	4.76
	60–69	56,789	31.88	48,469	85.35	3092	5.44	3052	5.37	2176	3.83
	70–79	63,327	35.55	53,165	83.95	3862	6.10	3743	5.91	2557	4.04
	80–89	21,098	11.84	17,331	82.15	1286	6.10	1616	7.66	865	4.10
	90–120	82	0.05	60	73.17	3	3.66	16	19.51	3	3.66
Oxford Hip Score											
	Severe	102,169	57.36	88,415	86.54	4307	4.22	6454	6.32	2993	2.93
	Moderate–severe	56,480	31.71	46,544	82.41	4059	7.19	3126	5.53	2751	4.87
	Mild–moderate	16,138	9.06	12,641	78.33	1295	8.02	1032	6.39	1170	7.25
	Satisfactory	1696	0.95	829	48.88	459	27.06	118	6.96	290	17.10
Sex											
	Male	66,951	37.59	56,438	84.30	3810	5.69	3888	5.81	2815	4.20
	Female	98,513	55.30	82,738	83.99	5729	5.82	6092	6.18	3954	4.01
	Missing	12 651	7.10	10,585	83.67	680	5.38	867	6.85	519	4.10
	Not specified	14	0.01	13	92.86	0	0.00	0	0.00	1	7.14
Symptom period (yr)											
	<1	23,183	13.01	19,885	85.77	1162	5.01	1329	5.73	807	3.48
	1–5	120,317	67.54	101,766	84.58	6719	5.58	7135	5.93	4697	3.90
	6–10	20,664	11.60	17 003	82.28	1334	6.46	1339	6.48	988	4.78
	>10	12,562	7.05	9972	79.38	893	7.11	959	7.63	738	5.87
Multimorbidity											
	Yes	38,384	21.55	30,773	80.17	2642	6.88	2981	7.77	1988	5.18
	No	139,745	78.45	118,101	84.51	7577	5.42	7866	5.63	5301	3.79
Disease count											
	0	76,459	42.92	65,751	86.00	3906	5.11	4003	5.24	2799	3.66
	1	63,286	35.53	53,250	84.14	3671	5.80	3863	6.10	2502	3.95
	2	27,123	15.23	22,062	81.34	1825	6.73	1960	7.23	1276	4.70
	3	8393	4.71	6576	78.35	594	7.08	710	8.46	513	6.11
	4	2144	1.20	1606	74.91	170	7.93	223	10.40	145	6.76
	5	491	0.28	344	70.06	40	8.15	69	14.05	38	7.74
	≥6	233	0.13	185	79.40	13	5.58	19	8.15	16	6.87
Individual chronic disease											
	Heart disease	15,773	8.85	12,680	80.39	1057	6.70	1227	7.78	809	5.13
	Hypertension	68,546	38.48	57,297	83.59	4146	6.05	4299	6.27	2804	4.09
	Stroke	2409	1.35	1903	79.00	174	7.22	190	7.89	142	5.89
	Circulation	8490	4.77	6563	77.30	620	7.30	762	8.98	545	6.42
	Lung disease	14,642	8.22	11,759	80.31	961	6.56	1155	7.89	767	5.24
	Diabetes mellitus	16,374	9.19	13,227	80.78	1071	6.54	1238	7.56	838	5.12
	Kidney disease	3365	1.89	2657	78.96	233	6.92	290	8.62	185	5.50
	Nervous system disorders	1395	0.78	1053	75.48	116	8.32	136	9.75	90	6.45
	Liver disease	1061	0.60	842	79.36	73	6.88	92	8.67	54	5.09
	Cancer	9466	5.31	7833	82.75	587	6.20	623	6.58	423	4.47
	Depression	14,112	7.92	10,952	77.61	1047	7.42	1284	9.10	829	5.87
	Arthritis	128,464	72.12	107,377	83.59	7625	5.94	8063	6.28	5399	4.20
Year											
	2013–4	36,823	20.67	30,652	83.24	2203	5.98	2331	6.33	1637	4.45
	2014–5	37,163	20.86	31,102	83.69	2207	5.94	2309	6.21	1545	4.16
	2015–6	35,032	19.67	29,438	84.03	2040	5.82	2126	6.07	1428	4.08
	2016–7	36,625	20.56	30,910	84.40	2032	5.55	2170	5.92	151	0.41
	2017–8	32,486	18.24	27,672	85.18	1737	5.35	1911	5.88	1166	3.59

### Outcomes

Most patients (149,774; 84.1%) had overall improvement in QoL as measured by PCHC. However, 10,219 (5.7%) had unchanged QoL, 19,847 (6.1%) had a mixed outcome, and 7289 (4.1%) reported worse QoL. Table 1 summarises the characteristics of patients stratified by the PCHC outcome. A further break down of PCHC categories is shown in Supplementary Table S3 with Supplementaryl Figure S1 showing the number of comorbidities and PCHC outcome.

Patients with a severe OHS before surgery had a better PCHC outcome 86.5% of the time, with those having a satisfactory OHS had a worse QoL, with only 48.9% having a better outcome. As the symptom period increased, QoL outcome worsened with those with a short period of <1 yr having a better outcome of 85.8% compared with those with a symptom period of > 10 yr, who had a better outcome 79.3% of the time.

Over time, there was a gradual improvement in outcome for all patients (Table 1), with better PCHC category increasing year on year from 83.2% in 2013–4 to 85.2% in 2016–7. There was an overall median change of –10 in EQ-VAS, which, together with a gain percentage (those with a postoperative EQ-VAS higher than the preoperative EQ-VAS) of 66.4%, shows overall improvement in QoL (Supplementary Table S4). When looking at each EQ-5D domain, there was an overall improvement in each domain (Supplementary Table S5).

### Multimorbidity and quality of life gain

**Fig 3:**
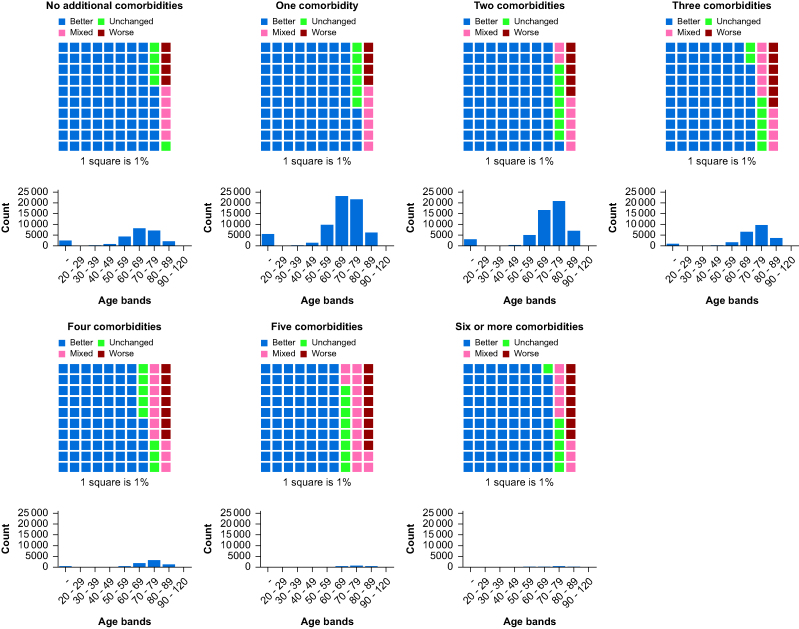
Waffle plot and age histogram. A waffle plot showing the proportion of patients per Pareto Classification of Health Change outcome cohorted into the number of comorbidities (excluding arthritis) with an age histogram.

Multimorbidity was present in 38,384 (21.6%) patients; these patients had a lower rate of better outcome of 80.2% than those without multimorbidity, who had a better quality measured by PCHC (84.5%). In addition, those with multimorbidity had a worse PCHC outcome of 5.2% than those without multimorbidity of 3.8% (Table 1). There was a stepwise decrease in gain of QoL as the number of diseases increased (Table 1; Supplementary Tables S3 and S4; Fig. 3; Supplementary Fig. S1).

**Fig 4:**
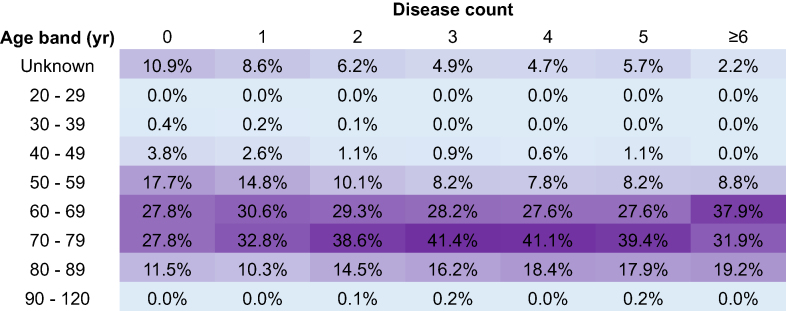
Heatmap. With the outcome ‘not better’ according to the Pareto Classification of Health Change (PCHC). ‘Not better’ is defined as the PCHC as unchanged, mixed, or worse. As the age increases, and as the number of comorbidities increases, the higher the percentage of patients with a ‘not better’ outcome.

Age and multimorbidity combine to reduce gain in QoL; as age and number of comorbidities increased, patient-reported outcome was worse (heatmap, Fig. 4). The effect on outcomes of advancing age and multimorbidity can also be demonstrated in the EQ-VAS change. The older the patients, the lower the percentage gain, and for patients >90 yr the median EQ-VAS change was 0 with a gain of 45.1%. As the number of comorbidities increased, the gain decreased; in the group with six or more comorbidities, the median gain in EQ-VAS was –6 and gain percentage was 57.9% (Supplementary Table S4).

### Adjusted association between multimorbidity and quality of life gain

The multivariable modelling looks at the adjusted association between the presence of multimorbidity and PCHC outcome of unchanged, mixed, or worse (summarised in Table 2). All associations were statistically significant. Patients with multimorbidity had 53% higher odds of having a poorer outcome (OR 1.53 [1.49–1.58]). Additional multivariable models are shown in Supplementary Table S6.

**Table 2:** Multivariable model.

Outcome	Pareto unchanged, mixed, worse		
	R2	0.034	
	C	0.601	
Variables		OR	95% CI
Multimorbidity			
	Present	1.53	1.49–1.58
Sex			
	Male	0.91	0.89–0.94
Oxford Hip Score category			
	Severe	ref	–
	Moderate to severe	1.47	1.43–1.51
	Mild to moderate	1.99	1.91–2.08
	Satisfactory	7.72	6.97–8.55
Symptom period (yr)			
	<1	0.86	0.82–0.9
	1–5	ref	–
	6–10	1.18	1.13–1.23
	>10	1.43	1.36–1.51
	Missing	1.09	0.94–1.26
Age bands (yr)			
	40–49	1.18	1.07–1.3
	50–59	1.07	1.02**–**1.11
	60–69	0.91	0.88–0.94
	70–79	ref	–
	80–89	1.17	1.12–1.22

### Exploratory analysis

When we repeated the core analysis excluding missing data on a per-domain basis, with this larger dataset (192 449 patients), the adjusted association between multimorbidity and poorer outcome was unchanged (OR 1.53 [1.46–1.55]).

## Discussion

The principal finding of this study is that multimorbidity, chronic disease burden, and age interact to reduce the QoL improvement associated with hip replacement. Although most patients experience a gain in QoL, a substantial minority (one in six) do not experience this benefit. To enhance shared decision-making for these patients, it is vital that they are made aware of this potential. In our modelling, we identified the importance of multimorbidity, age, and the number of comorbid diseases. Patients with more severe hip disease experience a greater gain in QoL, but those who experienced symptoms for a longer period before surgery have lesser gain in QoL. Multimorbidity was associated with less improvement in QoL.

Evidence suggests that no matter the baseline and multimorbidity pattern, most patients have improved QoL. There are a number of perioperative, patient, and psychosocial factors that impact outcome. When assessing perioperative factors, if nonoperative management has failed, there is improvement in pain and function in all groups who underwent total hip replacement and with lower preoperative PROMs leading to a greater improvement in QoL after surgery.^1^ We focused on patient and preoperative factors to model change in QoL. When looking at postoperative factors, a study looking at total hip replacement owing to primary osteoarthritis showed a moderate power of postoperative PROMs to predict future operation.^30^ If adverse events occur, such as re-operation owing to joint infection, periprosthetic fracture, or dislocation, there is a worse improvement in health-related quality of life (HRQoL).^31^^,^^32^ In addition, the role of anaesthesia and pain management has been shown to improve outcomes with local infiltration superior to neuraxial, and multimodal analgesia superior to unimodal practices.^1^

There is less evidence regarding patient factors. However, there is strong association between osteoarthritis and multimorbidity, with 50% of the over 65-yr age group with arthritis having significant cardiac, pulmonary, or mental health disease.^33^ The aging population means that the proportion of patients with significant comorbidity is increasing.^34^ Although multimorbid patients have a poorer QoL, prior studies have suggested they still experience a gain in QoL.^35^ Other studies have suggested that disease-specific QoL gain was lesser among multimorbid patients.^36^ Our findings align with this, suggesting that although most patients with multimorbidity experience a QoL gain, a substantial minority do not. Multimorbid patients wait longer for surgery and are less frequently offered surgical treatment.^37^ These findings have important implications for the increasing list of patients awaiting orthopaedic surgery, which exceeded 800,000 in March 2023.^38^ If individuals who are less likely to gain QoL after surgery can be reliably identified, then alternative treatments might be considered, and patients can be adequately informed of likely outcomes in the shared decision-making process. Of additional significance, our data and those of others^39^, ^40^, ^41^ show that the longer the surgical waiting time, the worse the outcome, which should be stressed as an important consideration for policy makers, healthcare practitioners, and organisations.

Several other patient-related factors can affect outcome, such as the geographical location of the operative hospital, but interestingly, not routinely by age, marital status, education level, or disposable income.^32^^,^^42^ BMI, sex, and comorbidity indices are conflicting with different studies showing differing results.^32^^,^^42^ When comorbidities are present, this explained the more modest improvement in HRQoL than would be anticipated.^32^ BMI was not a factor we looked at in the dataset as height and weight are not included in the collected PROMs variables, but low and high BMIs have been associated with poorer outcomes; not only did overweight and obese patients have a poorer outcome and increased risk of postoperative complications, those who were underweight had a poorer QoL than patients with normal BMI.^43^ This poorer outcome is not fully explained by BMI alone as low BMI infers poorly controlled underlying disease. BMI would be something to consider for future work looking at PROMs in this setting. Overall, not all characteristics that predispose to worse outcomes were captured in this dataset, thus the role of multimorbidity can be considered to have an association with outcomes, but the study cannot state that multimorbidity is in itself a causal factor in outcome determination because of this.

Our data showed a small improvement in outcomes over time, which aligns with previous work.^1^ Improvement in outcomes over time might be attributed to technical advances, such as computer navigation, minimally invasive approaches, robotic assistance, patient-specific instrumentation, implementation of novel pathways including fast-track surgical programmes with a focus on multifaceted treatment such as preoperative and postoperative rehabilitation and nutrition programmes.^1^^,^^31^^,^^32^

There are numerous strengths to this study. We analysed a large dataset, which included multiple years of surgeries, important variables (including age, complications, comorbidities), and QoL data that were all collected in a standardised manner. The hip replacement PROMs are collected in a standard manner that is repeatable over time, and includes preoperative and postoperative patient-reported QoL data. We prespecified a statistical analysis plan that we followed.

There were several weaknesses of the study. Firstly, this dataset captures around two-thirds of patients who underwent a hip replacement over the study period; however, the missing data owing to death is extremely small, with the remainder of the missing data unlikely to be only those with poor outcomes; the dataset is likely representative. In addition, there was 17.6% missingness which is, in part, owing to loss of follow-up. This can lead to over-representation of good outcomes in the complete patient data, in this instance leading to more positive results with patients with ‘not better’ or having died being underrepresented.^44^ As such, any bias resulting from this missingness means we under-report the incidence of poor outcomes. We did an exploratory analysis excluding individual domains where data were missing, rather than excluding the whole patient record if one piece of data was missing, and the findings were unchanged. The following risk factors for loss to follow-up were previously identified: (1) lower age, (2) deprivation, and (3) lower baseline QoL and OHS scores before and after surgery.^44^ We did not identify important differences between records with missing data and those with complete data. We were unable to account for surgical technique, chronic pain symptoms, and socioeconomic status in our cohort. There is evidence that surgical approach and use of cement affect QoL outcomes. Improved outcomes are reported in the posterior and lateral approaches; 95% of cases in the UK are lateral technique.^1^ Secondly, comorbidities were self-reported, which is prone to reporting bias with underrepresentation or incorrect representation. In addition, because of the binary nature of the comorbidity self-reporting, the severity of individual chronic diseases was not taken into account, which is important as more severe disease has a greater impact on outcomes.^45^^,^^46^ Thirdly, there are unmeasured covariables and potential unmeasurable confounders, particularly those not routinely measured by the dataset; the variables that influence outcome include biological factors such as surgery, anaesthesia, underlying patient factors and medical conditions, and also psychosocial influence, location, marital status, and education.^1^

### Conclusions

Hip replacement surgery is associated with improved QoL for most patients. However, a substantial minority (one in six) do not experience a gain in Quality of Life. The Quality of Life gain among patients with multimorbidity is more modest compared with patients without multimorbidity. Future research should explore features not captured by the PROMs dataset to enrich it. Important variables include operative approach, analgesia use, BMI, socioeconomic status, and detailed information related to perioperative complications. These data should inform shared decision-making conversations around joint replacement surgery.

## Authors’ contributions

Study design: AJF, JP, RP

Data collection and analysis: NV, AJF

Interpretation: NV, AJF

Writing the first draft of the manuscript: NV

Revision for important intellectual content and approval of the final version: all authors

## Funding

UK National Institute for Health and Care Research (NIHR) Doctoral Research Fellowship (DRF-2018-11-ST2-062 to AJF). The funding source had no role in the study design, data collection, analysis, interpretation, or writing the report.

## Declarations of interest

NV and JP report no conflicts of interest. AJF holds a National Institute for Health Research Doctoral Research fellowship (DRF-2018-11-ST2-062). RP has received honoraria, research grants, or both from Edwards Lifesciences, Intersurgical, and GlaxoSmithKline within the past 5 yr and holds editorial roles with the *British Journal of Anaesthesia* and the *British Journal of Surgery.*

## Data Availability

Data sharing on request.
