# Supplementary material for: Association between multimorbidity and quality of life after hip replacement surgery: analysis of routinely collected patient-reported outcomes
Source: Br J Anaesth. 2024 Nov 13;134(1):203–11. doi: 10.1016/j.bja.2024.08.037 (PMC11718361; doi:10.1016/j.bja.2024.08.037)
Supplement: Multimedia component 1 [file mmc1.docx]

**Supplementary File**

**Table S1**

|  |  | complete cases n(column%) | missing n(column %) | p-value |
| --- | --- | --- | --- | --- |
| Number |  | 178129 (82.39) | 38062 (17.61) |  |
| Year |  |  |  |  |
|  | 2013/2014 | 36823 (20.7) | 8123 (21.3) | 0.489 |
|  | 2014/2015 | 37163 (20.9) | 8708 (22.9) |  |
|  | 2015/2016 | 35032 (19.7) | 7572 (19.9) |  |
|  | 2016/2017 | 36625 (20.6) | 7935 (20.8) |  |
|  | 2017/2018 | 32486 (18.2) | 5724 (15) |  |
| Age Band |  |  |  |  |
|  | unspecified | 12651 ( 7.1) | 2524 (6.6) | 0.499 |
|  | 20 -29 | 5 ( 0.0) | 0 (0.0) |  |
|  | 30 -39 | 168 ( 0.1) | 20 (0.1) |  |
|  | 40 -49 | 2906 ( 1.6) | 447 (1.2) |  |
|  | 50 -59 | 21103 (11.8) | 3376 (8.9) |  |
|  | 60 -69 | 56789 (31.9) | 10469 (27.5) |  |
|  | 70 - 70 | 63327 (35.6) | 15148 (39.8) |  |
|  | 89 - 90 | 21098 (11.8) | 6057 (15.9) |  |
|  | >90 | 82 ( 0.0) | 21 (0.1) |  |
| Gender |  |  |  |  |
|  | male | 66951 (40.5) | 12412 (34.9) | 0.500 |
|  | female | 98513 (59.5) | 23123 (65.1) |  |
|  | not specified | 14 ( 0.0) | 3 (0.0) |  |
| Co-morbidity | |  |  |  |
|  | Heart Disease | 15773 ( 8.9) | 3497 (9.2) | 0.489 |
|  | Hypertension | 68546 (38.5) | 14497 (38.1) |  |
|  | Stroke | 2409 ( 1.4) | 592 (1.6) |  |
|  | Circulation | 8490 ( 4.8) | 2371 (6.2) |  |
|  | Lung Disease | 14642 ( 8.2) | 2994 (7.9) |  |
|  | Diabetes | 16374 ( 9.2) | 3679 (9.7) |  |
|  | Kidney Disease | 3365 ( 1.9) | 761 (2.0) |  |
|  | Nervous System Disorders | 1395 ( 0.8) | 316 (0.8) |  |
|  | Liver Disease | 1061 ( 0.6) | 225 (0.6) |  |
|  | Cancer | 9466 ( 5.3) | 2007 (5.3) |  |
|  | Depression | 14112 ( 7.9) | 3071 (8.1) |  |
|  | Arthritis | 128464 (72.1) | 25660 (67.4) |  |
| Preoperative living arrangement | |  |  |  |
|  | receiving assistance | 28514 (16.0) | 7398 (19.4) | 0.500 |
|  | no assistance | 148359 (83.3) | 29943 (78.7) |  |
|  | missing | 1256 ( 0.7) | 721 (1.9) |  |
| Postoperative living arrangement | |  |  |  |
|  | receiving assistance | 10371 ( 5.8) | 2727 (7.2) | 0.500 |
|  | no assistance | 166423 (93.4) | 34643 (91.0) |  |
|  | Missing | 1335 ( 0.7) | 692 (1.8) |  |
| Pre-op OHS score | |  |  |  |
|  | severe | 102169 (57.36) | 23287 (61.18) | 0.500 |
|  | moderate - severe | 56480 (31.71) | 10921 (28.69) |  |
|  | mild – moderate | 16138 (9.06) | 2929 (7.70) |  |
|  | satisfactory | 1696 (0.95) | 311 (0.82) |  |
|  | missing | 1646 (0.92) | 614 (1.61) |  |
|  | score median(IQR) | 18 (12) | 17 (12) |  |
| symptom period | |  |  |  |
|  | <1 | 23183 (13.01) | 5428 (14.26) | 0.500 |
|  | 1-5 | 120317 (67.54) | 24964 (65.59) |  |
|  | 6-10 | 20664 (11.60) | 3978 (10.45) |  |
|  | >10 | 12562 (7.05) | 2378 (6.25) |  |
|  | missing | 1403 (0.79) | 1314 (3.45) |  |
| number of comorbidities | |  |  |  |
|  | 0 | 25336 (14.22) | 6814 (17.90) | 0.500 |
|  | 1 | 67617 (37.96) | 13312 (34.97) |  |
|  | 2 | 52765 (29.62) | 10897 (28.63) |  |
|  | 3 | 22630 (12.70) | 4800 (12.61) |  |
|  | 4 | 7203 (4.04) | 1577 (4.14) |  |
|  | 5 | 1917 (1.08) | 452 (1.19) |  |
|  | >=6 | 661 (0.37) | 210 (0.55) |  |

# **Table S2 Top and Bottom 10 Pairs and Triads of Co-morbidities after excluding arthritis**

| 2 Diseases | |  |  | 3 Diseases |  |  |
| --- | --- | --- | --- | --- | --- | --- |
|  |  | n | % |  | n | % |
| Overall |  | 38352 | 21.530% |  | 10738 | 6.028% |
| top 10 |  |  |  |  |  |  |
| 1 | Hypertension\|Diabetes | 10090 | 5.664% | Heart Disease\|Hypertension\|Diabetes | 1759 | 0.987% |
| 2 | Heart Disease\|Hypertension | 6705 | 3.764% | Heart Disease\|Hypertension\|Lung Disease | 910 | 0.511% |
| 3 | Hypertension\|Lung Disease | 4519 | 2.537% | Hypertension\|Lung Disease\|Diabetes | 807 | 0.453% |
| 4 | Hypertension\|Depression | 3416 | 1.918% | Heart Disease\|Hypertension\|Circulation | 669 | 0.376% |
| 5 | Hypertension\|Cancer | 2227 | 1.250% | Hypertension\|Diabetes\|Depression | 655 | 0.368% |
| 6 | Hypertension\|Circulation | 2139 | 1.201% | Hypertension\|Lung Disease\|Depression | 540 | 0.303% |
| 7 | Lung Disease\|Depression | 962 | 0.540% | Hypertension\|Circulation\|Diabetes | 500 | 0.281% |
| 8 | Heart Disease\|Diabetes | 931 | 0.523% | Heart Disease\|Hypertension\|Depression | 404 | 0.227% |
| 9 | Hypertension\|Kidney Disease | 828 | 0.465% | Hypertension\|Diabetes\|Cancer | 392 | 0.220% |
| 10 | Heart Disease\|Lung Disease | 811 | 0.455% | Hypertension\|Circulation\|Depression | 334 | 0.188% |
| bottom 10 | |  |  |  |  |  |
| 10 | Nervous System\|Cancer | 28 | 0.016% | Heart Disease\|Stroke\|Circulation | 19 | 0.011% |
| 9 | Lung Disease\|Liver Disease | 27 | 0.015% | Lung Disease\|Kidney Disease\|Depression | 15 | 0.008% |
| 8 | Diabetes\|Nervous System | 26 | 0.015% | Diabetes\|Kidney Disease\|Depression | 13 | 0.007% |
| 7 | Stroke\|Cancer | 26 | 0.015% | Heart Disease\|Kidney Disease\|Cancer | 13 | 0.007% |
| 6 | Circulation\|Kidney Disease | 25 | 0.014% | Heart Disease\|Stroke\|Diabetes | 12 | 0.007% |
| 5 | Liver Disease\|Cancer | 20 | 0.011% | Heart Disease\|Kidney Disease\|Depression | 10 | 0.006% |
| 4 | Circulation\|Nervous System | 17 | 0.010% | Heart Disease\|Stroke\|Lung Disease | 10 | 0.006% |
| 3 | Heart Disease\|Liver Disease | 17 | 0.010% | Lung Disease\|Diabetes\|Kidney Disease | 7 | 0.004% |
| 2 | Kidney Disease\|Liver Disease | 14 | 0.008% | Heart Disease\|Circulation\|Kidney Disease | 6 | 0.003% |
| 1 | Stroke\|Kidney Disease | 5 | 0.003% | Circulation\|Diabetes\|Kidney Disease | 5 | 0.003% |
| overall: 50 pairing combinations | | | | overall: 61 triad combinations | | |

**Table S3 Clinically relevant variables stratified according to the PCHC outcome**

|  |  | Better |  | Unchanged |  | Mixed |  | Worse |  | total |
| --- | --- | --- | --- | --- | --- | --- | --- | --- | --- | --- |
| Overall |  | 149774 | 84.08% | 10219 | 5.74% | 10847 | 6.09% | 7289 | 4.09% | 178129 |
| Number of diagnoses including arthritis | |  |  |  |  |  |  |  |  |  |
| row n (row%) | 0 | 21872 | 86.33% | 1266 | 5.00% | 1302 | 5.14% | 896 | 3.54% | 25336 |
|  | 1 | 57991 | 85.76% | 3490 | 5.16% | 3635 | 5.38% | 2501 | 3.70% | 67617 |
|  | 2 | 44076 | 83.53% | 3184 | 6.03% | 3327 | 6.31% | 2178 | 4.13% | 52765 |
|  | 3 | 18311 | 80.91% | 1555 | 6.87% | 1669 | 7.38% | 1095 | 4.84% | 22630 |
|  | 4 | 5603 | 77.79% | 522 | 7.25% | 639 | 8.87% | 439 | 6.09% | 7203 |
|  | 5 | 1442 | 75.22% | 150 | 7.82% | 192 | 10.02% | 133 | 6.94% | 1917 |
|  | >=6 | 479 | 72.47% | 52 | 7.87% | 83 | 12.56% | 47 | 7.11% | 661 |
| Age Band | |  |  |  |  |  |  |  |  |  |
|  | * | 10634 | 84.06% | 703 | 5.56% | 799 | 6.32% | 515 | 4.07% | 12651 |
|  | 20-29 | 5 | 100.00% | 0 | 0.00% | 0 | 0.00% | 0 | 0.00% | 5 |
|  | 30 -39 | 142 | 84.52% | 7 | 4.17% | 13 | 7.74% | 6 | 3.57% | 168 |
|  | 40-49 | 2442 | 84.03% | 182 | 6.26% | 160 | 5.51% | 122 | 4.20% | 2906 |
|  | 50-59 | 17643 | 83.60% | 1212 | 5.74% | 1349 | 6.39% | 899 | 4.26% | 21103 |
|  | 60-69 | 47630 | 83.87% | 3323 | 5.85% | 3454 | 6.08% | 2382 | 4.19% | 56789 |
|  | 70-79 | 53455 | 84.41% | 3614 | 5.71% | 3771 | 5.95% | 2487 | 3.93% | 63327 |
|  | 80-89 | 17758 | 84.17% | 1172 | 5.56% | 1296 | 6.14% | 872 | 4.13% | 21098 |
|  | >90 | 65 | 79.27% | 6 | 7.32% | 5 | 6.10% | 6 | 7.32% | 82 |
| Diagnosis | |  |  |  |  |  |  |  |  |  |
|  | Heart Disease | 13356 | 84.68% | 880 | 5.58% | 945 | 5.99% | 592 | 3.75% | 15773 |
|  | Hypertension | 58102 | 84.76% | 3747 | 5.47% | 4041 | 5.90% | 2656 | 3.87% | 68546 |
|  | Stroke | 2010 | 83.44% | 129 | 5.35% | 159 | 6.60% | 111 | 4.61% | 2409 |
|  | Circulation | 7143 | 84.13% | 469 | 5.52% | 504 | 5.94% | 374 | 4.41% | 8490 |
|  | Lung Disease | 12397 | 84.67% | 830 | 5.67% | 852 | 5.82% | 563 | 3.85% | 14642 |
|  | Diabetes | 13899 | 84.88% | 868 | 5.30% | 1004 | 6.13% | 603 | 3.68% | 16374 |
|  | Kidney Disease | 2771 | 82.35% | 203 | 6.03% | 245 | 7.28% | 146 | 4.34% | 3365 |
|  | Nervous System Disorders | 1158 | 83.01% | 85 | 6.09% | 94 | 6.74% | 58 | 4.16% | 1395 |
|  | Liver Disease | 863 | 81.34% | 73 | 6.88% | 79 | 7.45% | 46 | 4.34% | 1061 |
|  | Cancer | 8052 | 85.06% | 506 | 5.35% | 560 | 5.92% | 348 | 3.68% | 9466 |
|  | Depression | 11915 | 84.43% | 816 | 5.78% | 811 | 5.75% | 570 | 4.04% | 14112 |
|  | Arthritis | 107773 | 83.89% | 7477 | 5.82% | 7952 | 6.19% | 5262 | 4.10% | 128464 |
| 10 commonest pairings | |  |  |  |  |  |  |  |  |  |
|  | Hypertension\|Diabetes | 8174 | 81.01% | 674 | 6.68% | 737 | 7.30% | 505 | 5.00% | 10090 |
|  | Heart Disease\|Hypertension | 5404 | 80.60% | 444 | 6.62% | 519 | 7.74% | 338 | 5.04% | 6705 |
|  | Hypertension\|Lung Disease | 3690 | 81.66% | 302 | 6.68% | 321 | 7.10% | 206 | 4.56% | 4519 |
|  | Hypertension\|Depression | 2704 | 79.16% | 244 | 7.14% | 282 | 8.26% | 186 | 5.44% | 3416 |
|  | Hypertension\|Cancer | 1908 | 85.68% | 128 | 5.75% | 119 | 5.34% | 72 | 3.23% | 2227 |
|  | Hypertension\|Circulation | 1685 | 78.78% | 159 | 7.43% | 174 | 8.13% | 121 | 5.66% | 2139 |
|  | Lung Disease\|Depression | 732 | 76.09% | 74 | 7.69% | 84 | 8.73% | 72 | 7.48% | 962 |
|  | Heart Disease\|Diabetes | 719 | 77.23% | 84 | 9.02% | 78 | 8.38% | 50 | 5.37% | 931 |
|  | Hypertension\|Kidney Disease | 685 | 82.73% | 53 | 6.40% | 48 | 5.80% | 42 | 5.07% | 828 |
|  | Heart Disease\|Lung Disease | 622 | 76.70% | 57 | 7.03% | 79 | 9.74% | 53 | 6.54% | 811 |
| 10 commonest triads | |  |  |  |  |  |  |  |  |  |
|  | Heart Disease\|Hypertension\|Diabetes | 1380 | 78.45% | 116 | 6.59% | 153 | 8.70% | 110 | 6.25% | 1759 |
|  | Heart Disease\|Hypertension\|Lung Disease | 706 | 77.58% | 62 | 6.81% | 82 | 9.01% | 60 | 6.59% | 910 |
|  | Hypertension\|Lung Disease\|Diabetes | 626 | 77.57% | 59 | 7.31% | 72 | 8.92% | 50 | 6.20% | 807 |
|  | Heart Disease\|Hypertension\|Circulation | 507 | 75.78% | 44 | 6.58% | 71 | 10.61% | 47 | 7.03% | 669 |
|  | Hypertension\|Diabetes\|Depression | 500 | 76.34% | 57 | 8.70% | 56 | 8.55% | 42 | 6.41% | 655 |
|  | Hypertension\|Lung Disease\|Depression | 415 | 76.85% | 40 | 7.41% | 41 | 7.59% | 44 | 8.15% | 540 |
|  | Hypertension\|Circulation\|Diabetes | 391 | 78.20% | 35 | 7.00% | 41 | 8.20% | 33 | 6.60% | 500 |
|  | Heart Disease\|Hypertension\|Depression | 315 | 77.97% | 30 | 7.43% | 36 | 8.91% | 23 | 5.69% | 404 |
|  | Hypertension\|Diabetes\|Cancer | 319 | 81.38% | 31 | 7.91% | 25 | 6.38% | 17 | 4.34% | 392 |
|  | Hypertension\|Circulation\|Depression | 244 | 73.05% | 34 | 10.18% | 31 | 9.28% | 25 | 7.49% | 334 |
| length of symptoms | | n(row%) |  |  |  |  |  |  |  |  |
|  | <1 | 19885 | 85.77% | 1162 | 5.01% | 1329 | 5.73% | 807 | 3.48% | 23183 |
|  | 1-5 | 101766 | 84.58% | 6719 | 5.58% | 7135 | 5.93% | 4697 | 3.90% | 120317 |
|  | 6-10 | 17003 | 82.28% | 1334 | 6.46% | 1339 | 6.48% | 988 | 4.78% | 20664 |
|  | >10 | 9972 | 79.38% | 893 | 7.11% | 959 | 7.63% | 738 | 5.87% | 12562 |
| Complications | |  |  |  |  |  |  |  |  |  |
|  | any complication suffered | 43909 | 79.11% | 3838 | 6.92% | 4431 | 7.98% | 3323 | 5.99% | 55501 |
| Allergy |  |  |  |  |  |  |  |  |  |  |
|  | Yes | 14701 | 81.44% | 1151 | 6.38% | 1264 | 7.00% | 936 | 5.19% | 18052 |
|  | Missing | 8380 | 76.85% | 795 | 7.29% | 956 | 8.77% | 773 | 7.09% | 10904 |
| Readmission | |  |  |  |  |  |  |  |  |  |
|  | Yes | 8356 | 72.01% | 908 | 7.82% | 1234 | 10.63% | 1106 | 9.53% | 11604 |
|  | Missing | 1191 | 79.83% | 91 | 6.10% | 126 | 8.45% | 84 | 5.63% | 1492 |
| further surgery | |  |  |  |  |  |  |  |  |  |
|  | Yes | 2516 | 63.96% | 349 | 8.87% | 512 | 13.01% | 557 | 14.16% | 3934 |
|  | Missing | 1192 | 78.89% | 101 | 6.68% | 121 | 8.01% | 97 | 6.42% | 1511 |
| urinary complication | |  |  |  |  |  |  |  |  |  |
|  | Yes | 16607 | 79.21% | 1456 | 6.94% | 1701 | 8.11% | 1203 | 5.74% | 20967 |
|  | Missing | 10074 | 78.99% | 844 | 6.62% | 1023 | 8.02% | 813 | 6.37% | 12754 |
| wound complication | |  |  |  |  |  |  |  |  |  |
|  | Yes | 10248 | 75.71% | 1064 | 7.86% | 1172 | 8.66% | 1052 | 7.77% | 13536 |
|  | Missing | 10567 | 79.84% | 872 | 6.59% | 1057 | 7.99% | 739 | 5.58% | 13235 |
| post-operative bleeding | |  |  |  |  |  |  |  |  |  |
|  | Yes | 5101 | 75.87% | 554 | 8.24% | 578 | 8.60% | 490 | 7.29% | 6723 |
|  | Missing | 12356 | 78.61% | 1065 | 6.78% | 1301 | 8.28% | 996 | 6.34% | 15718 |
| ten worst pairs with prevalence more than 0.05% | | |  |  |  |  |  |  |  |  |
|  | Heart Disease\|Stroke | 63 | 65.63% | 11 | 11.46% | 9 | 9.38% | 13 | 13.54% | 96 |
|  | Heart Disease\|Kidney Disease | 84 | 79.25% | 9 | 8.49% | 9 | 8.49% | 4 | 3.77% | 106 |
|  | Circulation\|Cancer | 102 | 83.61% | 7 | 5.74% | 7 | 5.74% | 6 | 4.92% | 122 |
|  | Hypertension\|Liver Disease | 105 | 85.37% | 6 | 4.88% | 9 | 7.32% | 3 | 2.44% | 123 |
|  | Hypertension\|Nervous System | 144 | 72.73% | 18 | 9.09% | 23 | 11.62% | 13 | 6.57% | 198 |
|  | Diabetes\|Cancer | 186 | 83.04% | 14 | 6.25% | 13 | 5.80% | 11 | 4.91% | 224 |
|  | Circulation\|Lung Disease | 170 | 72.65% | 17 | 7.26% | 29 | 12.39% | 18 | 7.69% | 234 |
|  | Circulation\|Diabetes | 197 | 75.77% | 15 | 5.77% | 26 | 10.00% | 22 | 8.46% | 260 |
|  | Heart Disease\|Cancer | 229 | 79.24% | 18 | 6.23% | 30 | 10.38% | 12 | 4.15% | 289 |
|  | Lung Disease\|Cancer | 248 | 80.26% | 25 | 8.09% | 21 | 6.80% | 15 | 4.85% | 309 |
| Pre-operative OHS score | |  |  |  |  |  |  |  |  |  |
|  | Severe | 88415 | 86.54% | 4307 | 4.22% | 6454 | 6.32% | 2993 | 2.93% | 102169 |
|  | moderate_severe | 46544 | 82.41% | 4059 | 7.19% | 3126 | 5.53% | 2751 | 4.87% | 56480 |
|  | mild_moderate | 12641 | 78.33% | 1295 | 8.02% | 1032 | 6.39% | 1170 | 7.25% | 16138 |
|  | Satisfactory | 829 | 48.88% | 459 | 27.06% | 118 | 6.96% | 290 | 17.10% | 1696 |

**Figure S1 Alluvial plot**


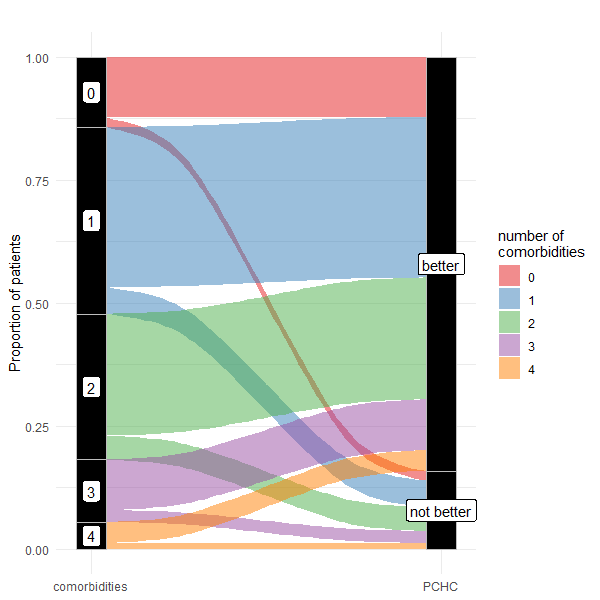


or more

**Table S4 Variables and EQVAS with Gain Percentage**

|  | | Median Pre (IQR) | Median Post (IQR) | Median EQ VAS Change | Number with post>pre ie gain | Gain percentage |
| --- | --- | --- | --- | --- | --- | --- |
| Overall |  | 70 (30.00) | 80 (20.00) | -10(25.00) | 118224 | 66.37% |
| Number of diagnoses including arthritis | |  |  |  |  |  |
|  | 0 | 75 (31) | 86 (20) | -10 (25) | 17008 | 67.13% |
|  | 1 | 70 (32) | 85 (15) | -10 (25) | 45957 | 67.97% |
|  | 2 | 70 (30) | 80 (20) | -10 (25) | 34809 | 65.97% |
|  | 3 | 60 (27) | 75 (25) | -10 (27) | 14513 | 64.13% |
|  | 4 | 58 (30) | 70 (30) | -10 (30) | 4400 | 61.09% |
|  | 5 | 50 (35) | 60 (30) | -10 (30) | 1154 | 60.20% |
|  | 6 | 50 (34.5) | 60 (25.25) | -5 (35) | 254 | 57.21% |
|  | 7 | 50 (26.5) | 55 (40) | -10 (35) | 56 | 58.95% |
|  | 8 | 45 (27.5) | 57 (37) | -10 (18.75) | 14 | 77.78% |
|  | 9 | 50 (35) | 75 (9) | -25 (40) | 4 | 80.00% |
|  | 10 | 70 (20) | 80 (20) | -20 (23) | 7 | 77.78% |
|  | 11 | 75 (40) | 85 (45) | -5 (20) | 14 | 56.00% |
|  | 12 | 80 (30) | 85 (20) | -5(21) | 34 | 52.31% |
| Age band |  |  |  |  |  |  |
|  | * | 70 (30.0) | 80 (20.00) | -10 (30.00) | 8807 | 69.62% |
|  | 20-29 | 40 (30.0) | 80 (15.00) | -40 (16.00) | 5 | 100.00% |
|  | 30 -39 | 65 (35.5) | 80 (22.25) | -15 (30.00) | 123 | 73.21% |
|  | 40-49 | 60 (40.0) | 80 (20.00) | -15 (33.00) | 2130 | 73.30% |
|  | 50-59 | 65 (35.0) | 80 (20.00) | -14 (30.00) | 15116 | 71.63% |
|  | 60-69 | 70 (30.0) | 80 (20.00) | -10 (25.00) | 39037 | 68.74% |
|  | 70-79 | 70 (30.0) | 80 (20.00) | -10 (21.00) | 40177 | 63.44% |
|  | 80-89 | 70 (30.0) | 78 (23.00) | -8 (26.00) | 12792 | 60.63% |
|  | >90 | 60 (25.0) | 70 (30.00) | 0 (34.75) | 37 | 45.12% |
| Co-morbidity |  |  |  |  |  |  |
|  | Heart Disease | 62 (29.00) | 75 (25) | -10 (29) | 9871 | 62.58% |
|  | Hypertension | 70 (30.00) | 80 (22) | -10 (25) | 45030 | 65.69% |
|  | Stroke | 60 (35.00) | 70 (27) | -10 (30) | 1506 | 62.52% |
|  | Circulation | 57 (33.75) | 70 (30) | -10 (32) | 5333 | 62.82% |
|  | Lung Disease | 60 (32.00) | 75 (25) | -10 (30) | 9097 | 62.13% |
|  | Diabetes | 64 (30.00) | 75 (26) | -10 (27) | 10400 | 63.52% |
|  | Kidney Disease | 60 (33.00) | 73 (26) | -10 (30) | 2061 | 61.25% |
|  | Nervous System Disorders | 60 (35.00) | 70 (30) | -10 (30) | 857 | 61.43% |
|  | Liver Disease | 60 (37.00) | 70 (27) | -10 (32) | 673 | 63.43% |
|  | Cancer | 68 (30.00) | 80 (25) | -10 (26) | 6048 | 63.89% |
|  | Depression | 51 (35.00) | 70 (30) | -10 (32) | 9193 | 65.14% |
|  | Arthritis | 70 (30.00) | 80 (20) | -10 (25) | 85111 | 66.25% |
| 10 commonest pairings | |  |  |  |  |  |
|  | Hypertension\|Diabetes | 60 (29) | 75 (25) | -10 (27) | 6401 | 63.44% |
|  | Heart Disease\|Hypertension | 60 (25) | 75 (25) | -10 (28) | 4231 | 63.10% |
|  | Hypertension\|Lung Disease | 60 (29) | 75 (25) | -10 (29) | 2875 | 63.62% |
|  | Hypertension\|Depression | 50 (31) | 70 (30) | -10 (30) | 2277 | 66.66% |
|  | Hypertension\|Cancer | 70 (30) | 80 (20) | -10 (25) | 1465 | 65.78% |
|  | Hypertension\|Circulation | 60 (30) | 71 (25) | -10 (26.5) | 1389 | 64.94% |
|  | Lung Disease\|Depression | 50 (35) | 65 (30) | -10 (34) | 585 | 60.81% |
|  | Heart Disease\|Diabetes | 60 (32) | 70 (30) | -8 (28) | 544 | 58.43% |
|  | Hypertension\|Kidney Disease | 70 (30) | 80 (24) | -10 (26) | 527 | 63.65% |
|  | Heart Disease\|Lung Disease | 60 (35) | 70 (30) | -10 (26) | 484 | 59.68% |
| 10 commonest triads | |  |  |  |  |  |
|  | Heart Disease\|Hypertension\|Diabetes | 60 (35) | 70 (30) | -9 (30) | 1057 | 60.09% |
|  | Heart Disease\|Hypertension\|Lung Disease | 60 (30) | 70 (30) | -7 (30) | 529 | 58.13% |
|  | Hypertension\|Lung Disease\|Diabetes | 60 (35) | 70 (30) | -9 (25) | 468 | 57.99% |
|  | Heart Disease\|Hypertension\|Circulation | 50 (30) | 70 (30) | -10 (30) | 417 | 62.33% |
|  | Hypertension\|Diabetes\|Depression | 50 (35) | 65 (30) | -10 (35) | 395 | 60.31% |
|  | Hypertension\|Lung Disease\|Depression | 50 (35) | 65 (30) | -10 (35) | 332 | 61.48% |
|  | Hypertension\|Circulation\|Diabetes | 55 (30) | 70 (30) | -10 (31.5) | 305 | 61.00% |
|  | Heart Disease\|Hypertension\|Depression | 50 (37.25) | 68 (30) | -10 (34.25) | 259 | 64.11% |
|  | Hypertension\|Diabetes\|Cancer | 65 (30) | 75.5 (20) | -10 (29) | 249 | 63.52% |
|  | Hypertension\|Circulation\|Depression | 50 (35) | 80 (20) | -10 (35) | 203 | 60.78% |
| Preoperative OHS scores | |  |  |  |  |  |
|  | Severe | 60 (40) | 80 (25) | -11 (30) | 69934 | 68.45% |
|  | moderate-severe | 75 (23) | 85 (15) | -10 (20) | 36989 | 65.49% |
|  | mild - moderate | 80 (20) | 85 (13) | -5 (17) | 9509 | 58.92% |
|  | satisfactory | 89 (15) | 90 (15) | 0 (14) | 779 | 45.93% |
| Number of co-morbidities including arthritis | |  |  |  |  |  |
|  | 0 | 75 (31) | 86 (20) | -10 (25) | 17008 | 67.13% |
|  | 1 | 70 (32) | 85 (15) | -10 (25) | 45957 | 67.97% |
|  | 2 | 70 (30) | 80 (20) | -10 (25) | 34809 | 65.97% |
|  | 3 | 60 (27) | 75 (25) | -10 (27) | 14513 | 64.13% |
|  | 4 | 58 (30) | 70 (30) | -10 (30) | 4400 | 61.09% |
|  | 5 | 50 (35) | 60 (30) | -10(30) | 1154 | 60.20% |
|  | ≥6 | 50 (35) | 60 (34) | -6 (35) | 383 | 57.94% |

# **Supplementary Table S5**

Table showing each EQ-5D domain and the overall improvement in PRO after surgery.

|  | EQ-5D individual domains n(column %) | | | |  |  |  |  |  |  |  |  |
| --- | --- | --- | --- | --- | --- | --- | --- | --- | --- | --- | --- | --- |
| change in profile | Mobility | | | | Self-Care | | | | Anxiety | | | |
|  | presurgery | | postsurgery | | presurgery | | postsurgery | | presurgery | | postsurgery | |
| no | 13580 | 7.62% | 108399 | 60.85% | 81684 | 45.86% | 145095 | 81.46% | 106118 | 59.57% | 146866 | 82.45% |
| slight | 163966 | 92.05% | 69583 | 39.06% | 94635 | 53.13% | 32209 | 18.08% | 64088 | 35.98% | 28223 | 15.84% |
| extreme | 583 | 0.33% | 147 | 0.08% | 1810 | 1.02% | 825 | 0.46% | 7923 | 4.45% | 3040 | 1.71% |
| Total: | 178129 | 100.00% | 178129 | 100.00% | 178129 | 100.00% | 178129 | 100.00% | 178129 | 100.00% | 178129 | 100.00% |
| change in profile | Discomfort | | | | Activity | | | |  |  |  |  |
|  | presurgery | | postsurgery | | presurgery | | postsurgery | |  |  |  |  |
| no | 1922 | 1.08% | 101687 | 57.09% | 11368 | 6.38% | 102590 | 57.59% |  |  |  |  |
| slight | 103309 | 58.00% | 70565 | 39.61% | 134870 | 75.71% | 70336 | 39.49% |  |  |  |  |
| extreme | 72898 | 40.92% | 5877 | 3.30% | 31891 | 17.90% | 5203 | 2.92% |  |  |  |  |
| Total: | 178129 | 100.00% | 178129 | 100.00% | 178129 | 100.00% | 178129 | 100.00% |  |  |  |  |

**Table S6 Multivariable Models**

All the individual co-morbidities were associated with an increased odds of readmission/reoperation. Of these, nervous system disease was associated with a statistically significant 97% increased odds of the reoperation/readmission, OR 1.97 (1.05 – 3.68). Stroke and kidney disease were the second and third co-morbidities of increased odds of the outcome at 68% (1.68[0.9-3.11]) and 64% (1.64[0.88 – 3.04]). In our chosen model, the odds ratios decreased as the co-morbidity increased as these are composites of the individual co-morbidities. The odds of having readmission/reoperation, and a worse PCHC outcome, showed a downward trend over the study period. (Table 2, S5). Males had a 19% increase in readmission/reoperation (OR 1.19 [1.14 - 1.24]), but a decreased odds of a worse PCHC outcome (Table 2, S5).

|  |  | model S1 | model S2 | model S3 |
| --- | --- | --- | --- | --- |
| Variables | Outcome | readmission/reoperation | Pareto to “not better” | Pareto to worse only |
|  | R2 | 0.036 | 0.116 | 0.133 |
|  | C | 0.627 | 0.69 | 0.768 |
| PreOp EQ5D | |  |  |  |
| **mobility** | |  |  |  |
|  | no problems | ref | ref | ref |
|  | slight problems | 1.14 (1.03 to 1.25) | 0.64 (0.6 to 0.67) | 0.6 (0.55 to 0.65) |
|  | extreme problems | 1.77 (1.36 to 2.29) | 0.63 (0.47 to 0.86) | 0.36 (0.14 to 0.89) |
| **selfcare** | |  |  |  |
|  | no problems | ref | ref | ref |
|  | slight problems | 1.12 (1.07 to 1.18) | 0.67 (0.65 to 0.69) | 0.58 (0.54 to 0.61) |
|  | extreme problems | 1.66 (1.43 to 1.94) | 0.73 (0.62 to 0.86) | 0.43 (0.28 to 0.66) |
| **activity** | |  |  |  |
|  | no problems | ref | ref | ref |
|  | slight problems | 1.01 (0.91 to 1.11) | 0.6 (0.57 to 0.63) | 0.56 (0.52 to 0.61) |
|  | extreme problems | 1.18 (1.05 to 1.31) | 0.41 (0.38 to 0.44) | 0.27 (0.24 to 0.31) |
| **discomfort** | |  |  |  |
|  | no problems | ref | ref | ref |
|  | slight problems | 0.87 (0.72 to 1.06) | 0.23 (0.21 to 0.26) | 0.5 (0.43 to 0.58) |
|  | extreme problems | 0.94 (0.77 to 1.15) | 0.13 (0.11 to 0.14) | 0.15 (0.13 to 0.18) |
| **anxiety** | |  |  |  |
|  | no problems | ref | ref | ref |
|  | slight problems | 1.05 (1 to 1.09) | 0.64 (0.62 to 0.67) | 0.61 (0.57 to 0.65) |
|  | extreme problems | 1.13 (1.03 to 1.24) | 0.42 (0.38 to 0.46) | 0.36 (0.3 to 0.44) |
| age band |  |  |  |  |
|  | 40 -49 | 0.9 (0.78 to 1.05) | 1.15 (1.04 to 1.28) | 1.39 (1.16 to 1.65) |
|  | 50 -59 | 0.86 (0.81 to 0.92) | 1.06 (1.02 to 1.11) | 1.29 (1.19 to 1.4) |
|  | 60 - 69 | 0.85 (0.81 to 0.89) | 0.89 (0.86 to 0.92) | 0.96 (0.9 to 1.02) |
|  | 70 -79 | ref | ref | ref |
|  | 80 -89 | 1.23 (1.16 to 1.3) | 1.2 (1.15 to 1.26) | 1.08 (1 to 1.18) |
| revision flag | |  |  |  |
|  |  | 2.6 (2.44 to 2.76) | 2.74 (2.61 to 2.88) | 2.69 (2.49 to 2.91) |
| Sex |  |  |  |  |
|  | male | 1.19 (1.14 to 1.24) | 0.89 (0.86 to 0.91) | 0.89 (0.85 to 0.94) |
|  | female | ref | ref | ref |
|  | missing | 2.77 (0.61 to 12.45) | 0.37 (0.05 to 2.89) | 1.58 (0.2 to 12.7) |
| Co-morbidity | |  |  |  |
|  | heart disease | 1.52 (0.82 to 2.79) | 0.57 (0.43 to 0.74) | 0.51 (0.34 to 0.79) |
|  | hypertension | 1.05 (1.01 to 1.1) | 1.07 (1.04 to 1.11) | 1.03 (0.98 to 1.09) |
|  | stroke | 1.68 (0.9 to 3.11) | 0.6 (0.45 to 0.8) | 0.6 (0.38 to 0.93) |
|  | circulation | 1.46 (0.79 to 2.69) | 0.75 (0.57 to 0.98) | 0.8 (0.52 to 1.22) |
|  | lung disease | 1.48 (0.8 to 2.72) | 0.59 (0.45 to 0.77) | 0.55 (0.36 to 0.84) |
|  | diabetes | 1.27 (0.69 to 2.34) | 0.58 (0.44 to 0.75) | 0.55 (0.36 to 0.85) |
|  | kidney disease | 1.64 (0.88 to 3.04) | 0.56 (0.42 to 0.74) | 0.51 (0.33 to 0.8) |
|  | nervous system | 1.97 (1.05 to 3.68) | 0.89 (0.66 to 1.19) | 0.76 (0.47 to 1.21) |
|  | liver disease | 1.71 (0.91 to 3.22) | 0.57 (0.42 to 0.77) | 0.46 (0.28 to 0.76) |
|  | cancer | 1.32 (0.71 to 2.43) | 0.47 (0.36 to 0.61) | 0.43 (0.28 to 0.66) |
|  | depression | 1.58 (0.86 to 2.91) | 1.03 (0.79 to 1.35) | 0.93 (0.61 to 1.43) |
| Disease count | |  |  |  |
|  | 0 | ref | ref | ref |
|  | 1 | 0.88 (0.48 to 1.62) | 2.43 (1.86 to 3.18) | 2.64 (1.73 to 4.03) |
|  | 2 | 0.74 (0.22 to 2.48) | 5.68 (3.34 to 9.66) | 7.24 (3.13 to 16.78) |
|  | 3 | 0.59 (0.1 to 3.67) | 11.77 (5.29 to 26.16) | 14.9 (4.22 to 52.69) |
|  | 4 | 0.44 (0.04 to 5.04) | 25.82 (8.83 to 75.5) | 41.85 (7.69 to 227.78) |
|  | 5 | 0.29 (0.01 to 6.28) | 40.21 (10.2 to 158.57) | 45.01 (5 to 405.16) |
|  | ≥6 | 0.02 (0 to 7.03) | 90.41 (9.28 to 880.62) | 232.41 (7.01 to 7702.57) |
| Preoperative OHS category | |  |  |  |
|  | satisfactory | 0.91 (0.72 to 1.14) | 0.67 (0.58 to 0.77) | 0.48 (0.4 to 0.59) |
|  | mild - moderate | 0.9 (0.82 to 0.98) | 0.61 (0.58 to 0.65) | 0.62 (0.57 to 0.68) |
|  | moderate - severe | 0.89 (0.84 to 0.94) | 0.77 (0.74 to 0.8) | 0.7 (0.66 to 0.75) |
|  | severe | ref | ref | ref |
| Year |  |  |  |  |
|  | 2013-14 | 0.98 (0.93 to 1.04) | 1.03 (0.98 to 1.07) | 1.07 (0.99 to 1.16) |
|  | 2014-15 | ref | ref | ref |
|  | 2015-16 | 0.9 (0.85 to 0.96) | 0.97 (0.93 to 1.01) | 1 (0.93 to 1.09) |
|  | 2016-17 | 0.89 (0.84 to 0.95) | 0.96 (0.92 to 1.01) | 1.04 (0.96 to 1.13) |
|  | 2017-18 | 0.88 (0.83 to 0.94) | 0.91 (0.87 to 0.96) | 0.93 (0.85 to 1.01) |
| Symptom period in years | |  |  |  |
|  | <1 | 0.99 (0.93 to 1.05) | 0.83 (0.79 to 0.86) | 0.81 (0.74 to 0.88) |
|  | 1-5 | ref | ref | ref |
|  | 6-10 | 1.05 (0.99 to 1.11) | 1.14 (1.09 to 1.19) | 1.16 (1.07 to 1.25) |
|  | >10 | 1.04 (0.96 to 1.12) | 1.21 (1.15 to 1.28) | 1.17 (1.06 to 1.28) |
|  | missing | 1.29 (1.07 to 1.56) | 1.07 (0.92 to 1.26) | 0.84 (0.62 to 1.13) |
| readmission/reoperation ref = yes | | |  |  |
|  |  | - | - | 2.88 (2.68 to 3.1) |
